# Long-Term Outcomes of Same Patient Eyes Treated with Pars Plana Vitrectomy in One Eye and Conventional Treatment in the Other for Complications of Proliferative Diabetic Retinopathy

**DOI:** 10.3390/jcm11185399

**Published:** 2022-09-14

**Authors:** Maria H. Berrocal, Luis Acaba-Berrocal, Alexandra M. Acaba

**Affiliations:** 1 Drs. Berrocal and Associates, San Juan, PR 00940, USA; 2 Illinois Eye and Ear Infirmary, University of Illinois Chicago, Chicago, IL 60612, USA

**Keywords:** pars plana vitrectomy, proliferative diabetic retinopathy, laser photocoagulation, vitreous hemorrhage, tractional retinal detachments

## Abstract

The purpose of this study was to evaluate the long-term, real-world outcomes of pars plana vitrectomy (PPV) for complications of proliferative diabetic retinopathy. A retrospective review involving 64 patients with proliferative diabetic retinopathy that underwent PPV in their worse-seeing eye were followed for a minimum of 8 years. The fellow eye underwent conventional treatment. Patients were divided into two groups by age: patients younger than 50 years of age and patients older than 50. In the younger than 50 group, 89% of vitrectomized eyes had improved visual acuity (VA) while 3.6% had decreased VA. A total of 14% of vitrectomized eyes required additional laser and 11% required reoperations. In the conventional treatment eyes, 25% had improved VA while 68% had decreased VA (*p* < 0.05). A total of 72% required additional laser and 60% required PPV. In the older than 50 group, 86% of vitrectomized eyes had VA improvement and 3% had decreased VA. A total of 8% required laser and 8% required reoperations. In the conventional treatment eyes, 30% improved VA and 48% had decreased VA (*p* < 0.05). Additional procedures required included laser in 70% and PPV in 17%. In both age groups, eyes that underwent PPV had better final visual outcomes than eyes that received conventional treatment for PDR.

## 1. Introduction

The prevalence of diabetes continues to increase globally. Worldwide, there are over 460 million patients with diabetes and that number is projected to increase over the next decade [1]. In the United States, around 50% of the population is estimated to suffer from prediabetes or diabetes [2,3]. The leading cause of visual loss in patients with diabetes is diabetic retinopathy, and specifically, proliferative diabetic retinopathy (PDR) [2]. Unfortunately, over 60% of patients with diabetes will develop PDR, with a significant percentage experiencing severe vision loss [4]. Standard treatment for PDR without associated vitreous hemorrhage (VH), tractional retinal detachments (TRD), or combined tractional and rhegmatogenous retinal detachment (TRRD) involves panretinal photocoagulation (PRP) and/or intravitreal anti-Vascular Endothelial Growth Factor (VEGF) injections. Panretinal photocoagulation reduces the risk of severe vision loss in PDR, but despite treatment, 15% of eyes still require vitrectomy [5,6]. Similarly, anti-VEGF treatment has been advocated for severe NPDR and PDR, however, 33% of eyes treated with anti-VEGF who are lost to follow-up develop TRD [7]. Missed appointments are frequent among patients with diabetes, with over 50% of patients being lost to follow up for 6 months or more [8]. In the United States, monthly anti-VEGF injections might not be a feasible option for patients due to cost, as more than 2 million patients with diabetes have no health insurance, as well as due to frequency of treatment [9]. Therefore, cost-effective treatments that provide long-term stability of DR and PDR are paramount.

One of the major complications and causes of severe vision loss in patients with PDR is TRDs, and repair of TRD is the most common indication for pars plana vitrectomy (PPV) in eyes with PDR [10]. TRDs tend to be more frequent and severe in younger patients with PDR due to the attached posterior hyaloid. Posterior vitreous detachment (PVD) is protective not only against TRD development but also against progression of diabetic retinopathy [11]. Multiple studies have demonstrated the ability of vitrectomy to improve visual outcomes and stabilize eyes long-term in eyes with TRDs, with reported retinal attachment rates of 98% and visual acuity improvement in 75–90% of cases [12,13,14,15]. However, there is a paucity of data comparing the real-world, long-term outcomes of PPV to conventional treatment in patients with proliferative diabetic retinopathy. In this study, we retrospectively compare the long-term outcomes of patients with PDR who had their worse-seeing eye treated with vitrectomy for VH, TRD, or TRRD and their better seeing eye treated initially with conventional treatment such as PRP.

## 2. Materials and Methods

This is a retrospective review of patients with diabetes mellitus who presented with complications of PDR requiring pars plana vitrectomy including VH, TRD, or TRRD in one eye and had conventional treatment in their other eye between 2003–2006. Conventional treatment included panretinal photocoagulation for PDR and focal laser to microaneurysms for macular edema initially. Anti-VEGF was not utilized initially as treatment for macular edema as it was not the standard of care at the time. Once anti-VEGF became the standard of care for macular edema, it replaced focal laser, and patients were treated with anti-VEGF if they developed center-involving macular edema. No grid focal treatment was performed. Both eyes were treated as needed during the follow-up period based on the standards of care at the time. Treatments during the follow-up period included added PRP laser for increased proliferation or mild VH, focal laser/anti-VEGF to microaneurysms for circinate exudates and center involving macular edema, phacoemulsification with intraocular lens implantation for visually impairing cataract progression, and PPV for VH, TRD, or TRRD. Patients with PDR in both eyes were included. Exclusion criteria included monocular patients, patients that had indications for PPV in both eyes at presentation, or those who had significant cataract requiring combined cataract extraction/vitrectomy. All patients completed at least 8 years of follow-up. Demographics, best corrected visual acuity (BCVA) outcomes at last follow-up, complications, and number and type of treatments required during the follow-up period were evaluated. Data were analyzed separately for patients younger than or equal to 50 years of age and patients older than 50 years at presentation. In each group, the eye that underwent initial vitrectomy was compared to the other eye of the patient, the conventional treatment eye. The study was conducted according to the tenets of the Declaration of Helsinki. Visual acuity was recorded and converted to the logarithm of the minimal angle of resolution (logMAR) for statistical analysis. A comparison of continuous variables was performed utilizing a student t-test, while categorical data were analyzed using a Chi squared test. A *p* value of <0.05 was used to determine statistical significance. Analysis and descriptive statistics were calculated using Microsoft Excel (Redmond, WA, USA) software version 16.64.

## 3. Results

A total of 64 patients met the inclusion criteria and were included in the study.

### 3.1. Younger Than 50 Years

A total of 28 patients younger than 50 years of age were included. Average age was 40 years (range 21–49) and mean self-reported duration of diabetes was 18 years (range 10–30). Mean hemoglobin A1c was 10.7 (range 8–14). Average follow-up was 9.5 years (range 8–17). All patients were bilaterally phakic. Nineteen eyes (68%) were lost to follow-up for 6 months or more during the 8-year period. (Table 1).

In the eye initially treated with vitrectomy, indications for surgery included TRD in 15 eyes (54%), TRRD in two eyes (7%), and VH in 11 eyes (39%). A total of 17 eyes (60%) underwent PPV with hyaloid removal and laser photocoagulation and 11 eyes (40%) had PPV with laser photocoagulation and gas tamponade with C3F8. Mean pre-op BCVA was 20/200 (range 20/80–LP) and final BCVA was 20/80 (range 20/30–LP) (*p* < 0.05). At last follow-up, BCVA improved in 25 eyes (89%) and decreased in one eye (3.6%). Two eyes (7%) had final BCVA of hand motion (HM) or less. Procedures required during the follow-up period included additional laser in four eyes (14%) (three for active neovascularization, one for macular edema) and re-operation with PPV in three eyes (11%) for RRD in one eye (with C3F8 tamponade) and recurrent VH (no tamponade used) in two eyes. Cataract progression occurred in 11 eyes (40%) with six eyes (21%) undergoing phacoemulsification with intraocular lens (phaco/IOL) implantation for visually impairing cataracts. Two eyes (7%) developed glaucoma, with one eye requiring filtering surgery (3.5%). On average, additional procedures required occurred at 25.4 months (range 1 m–11years). (Table 2) Comparing BCVA between eyes that required additional procedures and eyes that did not, there was no significant difference between them (20/80 vs. 20/100, respectively, *p* = 0.90).

In the eye treated with conventional therapy, average initial BCVA was 20/80 (range 20/20–HM), while mean final BCVA was 20/400 (range 20/30–NLP) (*p* < 0.05). At last follow-up, BCVA improved in seven eyes (25%) and decreased in 19 eyes (68%). Ten eyes (35%) had a final BCVA of HM or less. Procedures required during the follow-up period included additional laser in 20 eyes (72%), in 17 for PDR and in three for macular edema, anti-VEGF with bevacizumab for macular edema in two eyes, and PPV in 17 eyes (60%). Indications for surgery during the follow-up period were TRD in 12 eyes (43%), TRRD in four eyes (14%), and VH in one eye (3.6%). Four eyes (14%) developed glaucoma and seven eyes (25%) had cataract progression with three eyes undergoing phaco/IOL (11%). On average, additional procedures required occurred at 28.3 months (range 2 m–0 years). There was no difference in timing of additional procedures between the vitrectomized and conventional therapy eyes (*p* = 0.68). Statistically significant differences (*p* < 0.05) were present between the initially vitrectomized eyes and the conventional therapy eyes in terms of initial and final BCVA, percent improvement in BCVA and percent worsening in BCVA, and additional procedures required. At final follow-up, no patients were scheduled to receive specific treatments and all patients were scheduled to follow at 6–12-month intervals.

### 3.2. Older Than 50 Years

A total of 36 patients 50 years or older were included. Mean age was 60 (range 51–72) and average self-reported duration of diabetes was 18 years (range 4–30). Average hemoglobin A1c was 9.4 (range 6.4–12). Mean follow-up was 9.3 years (range 8–14). Three patients were bilaterally pseudophakic and 33 patients were bilaterally phakic. Nineteen eyes (53%) were lost to follow up for 6 months or more during the 8-year period.

In the eye initially treated with vitrectomy, indications for surgery included TRD in 10 eyes (28%), TRRD in two eyes (5%), and VH in 24 eyes (67%). A total of 26 eyes (72%) underwent PPV with hyaloid removal and laser photocoagulation and 10 eyes (28%) had PPV with laser photocoagulation and gas tamponade with C3F8. Mean pre-op BCVA was 4/200 (range 20/200–LP) and final BCVA was 20/80 (range 20/40–HM) (*p* < 0.05). At last follow-up, BCVA improved in 31 eyes (86%) and decreased in one eye (3%). Five eyes (14%) had final BCVA of count fingers (CF) or less. Procedures required during the follow-up period included additional laser in three eyes (8.3%), in two eyes for progressive neovascularization and in one eye for macular edema, anti-VEGF with bevacizumab for macular edema in one eye, and re-operation with PPV with laser and no tamponade for rebleeding in three eyes (8.3%). Visually significant cataracts requiring phaco/IOL occurred in 16 eyes (44%), and five eyes (14%) developed glaucoma. On average, additional procedures required occurred at 32.1 months (range 1 m–4years) (Table 3). Comparing BCVA between eyes that required additional procedures and eyes that did not, there was no significant difference between them (20/80 vs. 20/125 respectively, *p* = 0.86).

In the eye treated with conventional therapy, average initial BCVA was 20/100 (range 20/25–20/400), while final mean BCVA was 20/200 (range 20/30–NLP). At last follow-up, BCVA improved in 11 eyes (30.5%) and decreased in 17 eyes (48%). Thirteen eyes (36%) had BCVA of 5/200 or less. Procedures required during the follow-up period included additional laser in 25 eyes (69%), with 20 eyes requiring laser for PDR and five eyes for macular edema, anti-VEGF with bevacizumab in one eye for macular edema, and PPV in six eyes (17%) for TRD. Six eyes (17%) developed glaucoma and six eyes (17%) had cataract progression, with five eyes (14%) undergoing phaco/IOL for visually significant cataracts. One eye required enucleation for phthisis. On average, additional procedures required occurred at 24.9 months (range 2 m–7 years). There was no difference in timing of additional procedures between the vitrectomized and conventional therapy eyes (*p* = 0.48). Statistically significant differences (*p* < 0.05) were present between the initially vitrectomized eyes and the conventional therapy eyes in terms of initial and final BCVA, percent improvement in BCVA and percent worsening in BCVA, and additional procedures required. At final follow-up, no patients were scheduled to receive specific treatments and all patients were scheduled to follow at 6–12-month intervals.

## 4. Discussion

Understanding the real-world, long-term outcomes of vitrectomy surgery compared to standard nonsurgical treatment for PDR has the potential to improve visual outcomes, proliferative diabetic retinopathy disease burden, and overall cost related to PDR treatment. The key findings of this study are (1) initial treatment with vitrectomy was associated with greater improvement of visual acuity and less additional procedures at final follow-up compared to eyes treated with conventional therapy, and (2) younger patients treated with conventional therapy had greater rates of needing subsequent PPV compared to older patients.

Overall, initial vitrectomy surgery was associated with significant improvement in final visual acuity in both the younger and older age groups compared to conventional therapy. These findings are prescient since the eyes undergoing initial vitrectomy had more advanced disease relative to the other eye, yet they had better final visual acuities. Given the long-term follow-up period of at least 8 years in these patients, initial vitrectomy surgery seemed to have provided a stabilizing and protective effect to the eye against the progression of PDR.

In our study, the younger group of patients that were treated with conventional therapy required more PPV interventions compared to the older group during the follow-up period. This is possibly related to the status of the hyaloid, with older patients more likely to have a complete PVD. Although, multiple eyes initially treated with vitrectomy required additional procedures during the follow-up period, there was no difference in final BCVA between those that did not require additional procedures and those that did, indicating that the additional procedures were likely not providing further baseline VA gains compared to initial PPV. Studies have demonstrated that progression of DR is dependent on the status of the vitreous [11]. In a study of 403 eyes of patients with DR followed for 3 years, eyes that had a complete PVD showed no progression of retinopathy [11]. In eyes that had no PVD, progression of DR occurred in 44% while eyes that had a partial PVD had 100% progression in retinopathy. Similarly, in patients with DR, a PVD is independently associated with reduced incidence of macular edema compared to eyes without a PVD [16]. When determining ideal treatments for patients, the status of the hyaloid needs to be considered. Eyes without a PVD with advanced PDR may benefit from vitrectomy with removal of the hyaloid and photocoagulation, as vitrectomized eyes tend to remain stable long term.

The ideal time to perform PPV in eyes with complications of DR remains controversial. The only large study that evaluated the benefits of performing earlier vitrectomy vs. observation was the Diabetic Retinopathy Vitrectomy Study (DRVS) in 1985 [17]. In the study arm of eyes with advanced PDR without hemorrhage, vitrectomized eyes showed better visual acuity outcomes at 4 years, with 44% of eyes having a visual acuity of 10/20 or better compared to 28% in eyes undergoing conventional treatment [17]. This improvement with vitrectomy was more significant in patients with type 1 diabetes and eyes with more advanced disease. Despite advancements in treatments and vitrectomy technology since 1985, the DRVS findings are similar to those seen in our study, with the vitrectomized eyes having better visual acuity outcomes compared to eyes undergoing conventional treatment.

One limitation was the number of patients lost to follow-up. Obeid et al., have shown the frequency and detrimental effects of missed appointments in diabetic patients, particularly in eyes treated with anti-VEGF vs. PRP [7]. Nevertheless, between 58% and 68% of patients in our study were lost to follow-up for a period of 6 months or more. The eyes that were lost to follow-up for a period of over a year had worse BCVA compared to the eyes not lost to follow-up, underscoring the detrimental effects of poor compliance with follow-up and treatments in diabetic patients. Lost to follow-up can involve many reasons, including economic challenges, insurance status, illnesses, transportation costs, health literacy, work concerns, among others.

The cost of diabetes care is significant and worldwide many patients are challenged to keep numerous appointments and pay for costly ophthalmological treatments, including anti-VEGF injections. Severe complications of diabetes affect socioeconomically challenged patients disproportionately, resulting in poorly insured and uninsured patients exhibiting more end-stage renal disease and blindness. In the US, 25% of all healthcare expenditures go to the care of diabetes and its complications [18]. In a comparison of the cost of vitrectomy, PRP, and intravitreal ranibizumab in the US for 2 years of treatment, the cost of early vitrectomy was significantly lower than the cost of anti-VEGF, and the treatment burden with vitrectomy was reduced [19]. Overall, vitrectomy is an attractive treatment modality for PDR complications that stabilizes eyes long-term and reduces the treatment burden of diabetic patients during their productive years.

Our study shows real-world long-term results of eyes of patients with diabetes treated for complications of DR. During the 8-year follow-up period, many patients missed appointments, failed to come in expeditiously when visual acuity decreased, and presented with advanced disease despite having photocoagulation. Patients that were lost to follow-up for a period of time were included in the analysis and were found to have significantly worse BCVA, underscoring what happens in real-world retina practices and not in patients closely followed within a study protocol. The results of this study demonstrate the stabilizing effect of vitrectomy on PDR disease progression. Strengths of this study include the long-term follow-up data of at least 8 years. Weaknesses include the retrospective nature, patients lost to follow-up for periods over 6 months, and the lack of hyaloid status assessment. It is important to note that patients lost to follow-up for a period of time were included in the analysis, representing a real-world scenario, but this confounds final BCVA between groups and represents a limitation for the study. Future studies should investigate outcomes based on hyaloid status rather than age, since these would provide more information regarding the ideal timing of vitrectomy in eyes with advanced PDR to prevent complications.

## Figures and Tables

**Table 1:** Patient demographics.

	Age Less than 50 Years	Age Greater than 50 Years	*p* Value
**Number of Patients**	28	36	-
**Age in years (range)**	40 (21–49)	60 (51–72)	-
**Duration of diabetes in years (range)**	18 (10–30)	18 (4–30)	0.55
**Mean Hemoglobin A1c (range)**	10.7 (8–14)	9.5 (6.4–12)	0.46
**Indications for Vitrectomy (%)**			
TRD	15 (54)	10 (28)	0.04
TRRD	2 (7)	2 (5)	0.73
VH	11 (39)	24 (67)	0.03
**Lens Status (%)**			
Phakic	0	33	
Pseudophakic	28 (100)	3 (8.3) *	<0.001
**Vitrectomy procedure performed (%)**			
Vitrectomy with endolaser	17 (60)	26 (72)	0.31
Vitrectomy with endolaser and gas tamponade	11 (40)	10 (28)	0.32
**Average follow-up in years (range)**	9.5 (8–17)	9.3 (8–14)	0.84

TRD: tractional retinal detachment, TRRD: combined tractional and rhegmatogenous retinal detachment, VH: vitreous hemorrhage. * All 3 pseudophakic patients were bilaterally pseudophakic.

**Table 2:** Results, younger than 50 years.

	Initial Vitrectomy	Conventional Therapy	*p* Value
**Initial mean BCVA (range)**	20/200 (20/80-LP)	20/80 (20/20-HM)	0.03
**BCVA at last follow-up (range)**	20/80 (20/30-LP)	20/400 (20/30-NLP)	0.01
**Eyes with improvement in BCVA (%)**	25 (89)	7 (25)	<0.001
**Eyes with worsening BCVA (%)**	1 (3.6)	19 (68)	<0.001
**Additional procedures required (%)**			
Laser photocoagulation	4 (14)	20 (72)	<0.001
Vitrectomy	3 (11)	17 (60)	0.0001
Phaco/IOL	6 (21)	3 (10)	0.26
Glaucoma surgery	1 (3.5)	0	-
Anti-VEGF	0	2 (7)	-
**Average time to additional procedure in months**	25.4	28.3	0.68
**Complications (%)**			
Cataract progression	11 (40)	7 (25)	0.24
Glaucoma development	2 (7)	4 (14)	0.40

BCVA: best corrected visual acuity.

**Table 3:** Results, older than 50 years.

	Initial Vitrectomy	Conventional Therapy	*p* Value
**Initial mean BCVA (range)**	4/200 (20/200-LP)	20/100 (20/25–20/400)	0.01
**BCVA at last follow-up (range)**	20/80 (20/40-HM)	20/200 (20/30-NLP)	0.035
**Eyes with improvement in BCVA (%)**	31 (86)	11 (30.5)	<0.001
**Eyes with worsening BCVA (%)**	1 (3)	17 (48)	<0.001
**Additional procedures required (%)**			
Laser photocoagulation	3 (8.3)	25 (69)	<0.001
Vitrectomy	3 (8.3)	6 (17)	0.27
Phaco/IOL	16 (44)	6 (17)	0.014
Glaucoma surgery	5 (14)	5 (14)	-
Enucleation	0	1	-
Anti-VEGF	1	1	-
**Average time to additional procedure in months**	32.1	24.9	0.48
**Complications (%)**			
Cataract progression	16 (44)	7 (17)	0.013
Glaucoma development	5 (14)	7 (17)	0.73

## Data Availability

Data is contained within the article.
